# Inter-Reader Agreement in LR-TRA Application and NLR Association in HCC Patients Treated with Endovascular vs. Ablative Procedures

**DOI:** 10.3390/cancers17030492

**Published:** 2025-02-01

**Authors:** Davide Giuseppe Castiglione, Annamaria Porreca, Daniele Falsaperla, Federica Libra, Emanuele David, Roberta Maiuzzo, Mirko Domenico Castiglione, Cristina Mosconi, Stefano Palmucci, Pietro Valerio Foti, Antonio Basile, Massimo Galia

**Affiliations:** 1 Radiology Unit 1, Department of Medical Surgical Sciences and Advanced Technologies “GF Ingrassia”, University of Catania, University Hospital Policlinico “G. Rodolico-San Marco”, 95123 Catania, Italy; 2 Section of Radiology, Department of Biomedicine, Neuroscience and Advanced Diagnostics (BiND), University Hospital “Paolo Giaccone”, Via del Vespro 129, 90127 Palermo, Italy; 3 Department of Human Sciences and Promotion of the Quality of Life, San Raffaele Open University, Via di Val Cannuta, 247, 00166 Rome, Italy; annamaria.porreca@uniroma5.it; 4 University Hospital Policlinico “G. Rodolico-San Marco”, 95123 Catania, Italy; 5 Department of Radiology, Istituto di Ricovero e Cura a Carattere Scientifico-IRCCS, Azienda Ospedaliero-Universitaria di Bologna, Sant’Orsola-Malpighi Hospital, 40138 Bologna, Italy; 6 UOSD I.P.T.R.A., Department of Medical Surgical Sciences and Advanced Technologies “GF Ingrassia”, University of Catania, University Hospital Policlinico “G. Rodolico-San Marco”, 95123 Catania, Italy

**Keywords:** hepatocarcinoma, LI-RADS TRA, neutrophils-to-lymphocytes ratio (NLR), MWA, TAE, magnetic resonance imaging (MRI), inter-reader agreement, interventional radiology, tumor response, interventional oncology

## Abstract

Objectives: This study aimed to assess the performance of the LI-RADS tumor response algorithm in analyzing inter-reader agreement in patients with hepatocellular carcinoma (HCC) treated with Microwave Ablation (MWA) and Transarterial Embolization (TAE) and the relationship between inter-reader agreement and Neutrophils to Lymphocytes ratio dynamic variations at different time points to explore how inflammation influences tumor response and its interpretation on imaging. Methods: A retrospective analysis was conducted on 78 HCC patients treated with MWA or TAE. Two independent radiologists evaluated pre- and post-treatment imaging and assigned categories according to the LR-TRA. Inter-reader agreement was assessed with a focus on subgroup analysis considering the different locoregional treatments. NLR values, measured at baseline (T0), 72 h (T1), and 30 days post-procedure (T2), were compared with patients with concordant and discordant LR-TRA assessments. This analysis aimed to identify any association between NLR dynamics and inter-reader agreement on treatment response. Results: The inter-reader agreement in the LR-TRA application was “substantial” in the cases of MWA treatment evaluation (κ = 0.65), and “moderate” in the cases of TAE treatment evaluation (κ = 0.51). The differences in inter-reader agreement were found to be expressions of different levels of NLR mean values in the different time frames evaluated. Three days after treatment, NLR increased significantly in TAE groups. At 30 days, NLR had returned close to baseline levels but with NLR persisting higher in the TAE group. There was a statistically significant difference in NLR between the “mismatch” group (those with discrepant LR-TRA readings) and the “match” group at 3 days (*p* = 0.004) and late evaluation (30+ days). Conclusions: This study has shown that NLR levels can predict inter-reader discrepancies in LR-TRA assessment and may be translated into different levels of difficult imaging interpretation. Combining LR-TRA and NLR is promising for a more comprehensive assessment of tumor response and inflammatory dynamics.

## Simple Summary

The present study sought to investigate the factors contributing to inter-radiologist variability in assessing the efficacy of liver cancer treatments. It hypothesized that the type of treatment (Microwave Ablation or Transarterial Embolization) and patient immune response, as measured by the Neutrophils-to-Lymphocytes Ratio, could influence the variability in LI-RADS Treatment response (LR-TRA) algorithm application. The study aimed to determine if the NLR could serve as a predictive biomarker for inter-radiologist agreement on treatment effectiveness and to explore the impact of post-treatment immune response on imaging outcomes. The findings suggest that a combined assessment of imaging and blood-based biomarkers could enhance the accuracy and consistency of evaluating treatment efficacy in liver cancer patients, potentially leading to improved patient care.

## 1. Introduction

Hepatocellular Carcinoma (HCC) is a prevalent and deadly malignancy, representing a significant global health challenge. Managing HCC often involves locoregional image-guided treatments, and accurate assessment of treatment response is crucial for effective patient care [1,2]. The LI-RADS Treatment Response algorithm (LR-TRA) plays a pivotal role in this evaluation. However, the complexity of interpreting post-treatment imaging, considering also that expected imaging findings of post-treatment evaluation vary based on the type of locoregional treatment, necessitates a high degree of inter-reader reliability to ensure accurate and consistent assessment [3,4,5,6].

The diagnostic performance of the LR-TRA has been difficult to assess and estimate because several variables such as tumor size, radiologists’ experience, different locoregional therapies, pretreatment tumoral imaging behavior and imaging evaluation modalities may influence its value. In recent years, many investigations have been conducted to evaluate the accuracy and utility of the LI-RADS TR. These studies have focused on scrutinizing the inter-reader agreement and establishing a correlation between radiological findings and pathological outcomes after thermal ablative and intra-arterial embolic treatments. In particular, the validity of a treatment response algorithm should be demonstrated by a high degree of inter-reader reliability, given that interpretation significantly influences patient care decisions [7,8,9,10].

Notably, two recent studies conducted by Cools et al. and Chaudhry et al. revealed notably higher inter-reader agreement in the LR-TRA categorization process following thermal ablation, which includes radiofrequency ablation (RFA) and microwave ablation (MWA). These studies reported impressive inter-reader reliability rates of 90% and 95%, along with kappa values of 0.75 (Standard Error = ±0.09) and 0.71 (95% Confidence Interval: 0.59–0.84), respectively [7,8].

Conversely, the inter-reader agreement tends to be somewhat lower when comparing the LR-TRA categorization after non-radiation arterial-based therapies for HCC.

Specifically, Seo et al. reported that 78.6% of tumors underwent transarterial chemoembolization (TACE) and were assessed using computed tomography (CT) or magnetic resonance imaging (MRI). In the study by Shropshire et al. [6], all tumors were treated with transarterial embolization (TAE) and evaluated using MRI. The reported kappa values were 0.69 (for CT), 0.56 (for MRI), and 0.55, respectively [6,11]. However, the study by A. Razek et al. found no significant differences in interobserver agreement between patients treated by thermal ablation and those treated by TACE [12].

### Research Hot-Points

The disparity in inter-reader agreement between thermal ablation and arterial therapy may be unsurprising and logical, given that the expected imaging appearances post-ablation are comparatively simpler when contrasted with the often-intricate imaging characteristics observed following transcatheter arterial-based therapies. Nevertheless, as literature data are disharmonious and few comparative data exist to give evidence of inter-reader agreement differences in LR-TRA when applied to different LRTs, one of the main aims of this research is to explore differences in the inter-reader agreement using LR-TRA evaluating MWA and TAE in HCC cohort patients.

Different locoregional therapies (LRTs) may influence the diagnostic accuracy of post-treatment imaging findings. Recent research highlights the role of LRTs in initiating tumor-specific immune responses through oncolysis, a process that can determine treatment efficacy with unknown effects on post-treatment imaging evaluation accuracy [13,14].

Specifically, it has been observed that cell death after Radiofrequency Ablation (RFA) or Microwave Ablation (MWA) induces both innate and adaptive immune responses but limited empirical data are available concerning the impact of systemic inflammatory markers’ kinetics on the local progression of tumor [15,16]. This is relevant because they may pose questions about the unknown impact of the immune response elicited by different LRTs and its influence on radiologic evaluation of post-treatment response at LRTs.

The inflammatory immune response to LRTs has garnered increasing interest, particularly the role of biomarkers like the neutrophil-to-lymphocyte ratio (NLR) in predicting prognosis in solid tumors, including hepatocellular carcinoma (HCC). NLR has proven to be a reliable prognostic tool in HCC patients undergoing various treatments, with preoperative NLR predicting recurrence after ablative therapies [17,18,19,20].

Elevated NLR has been linked to disease progression and poor overall survival in HCC patients treated with transarterial chemoembolization (TACE). This may be attributed to neutrophils’ role in the tumor microenvironment, promoting tumorigenesis and recurrence [21,22,23,24,25].

Given the reliance on detecting vascular alterations in the radiological assessment of LRTs, there is a need to understand the interplay between NLR values and imaging accuracy in an immunomodulated environment. This study explores the correlation between inter-reader agreements in evaluating the performance of TRA applying inter-reader agreement evaluation and the NLR values in pre-treatment and post-treatment phases in two different scenarios, MWA and TAE.

## 2. Materials and Methods

This study adhered to ethical guidelines established in the Declaration of Helsinki (1975 and latest version) and the Health Insurance Portability and Accountability Act (HIPAA). Researchers conducted a retrospective analysis evaluating patients diagnosed with HCC who underwent endovascular embolization or percutaneous ablation between January 2019 and May 2023. Electronic medical records from the Hospital Center servers documented procedures during this timeframe. These records were cross-referenced with procedural reports archived within the Interventional Radiology service. A dedicated Interventional Radiologist notably performed all procedures at the same institution.

Data collection and cleaning were meticulous. They were overseen by an Interventional Radiologist with five years of experience, assisted by two Radiologists in training. Subsequently, anonymized data were stored in a secure electronic format.

This study employed strict inclusion criteria to ensure a homogeneous cohort. Eligible patients are presented with treatment-naïve HCC, of any etiology, measuring 2–5 cm, and treated with either MWA or TAE. Patients with any history of immunologic or oncologic disease were excluded. Essential for inclusion were pre-treatment MRI and laboratory tests at three specific time points (pre-procedural, 72 h, and 30 days post-procedure) and post-treatment MRI imaging [26,27,28].

Exclusion criteria encompassed individuals under 18 years old, those with incomplete treatment or follow-up information, patients with uninterpretable imaging studies not conforming to LI-RADS criteria, and those who did not meet the above inclusion criteria.

The decision to offer Interventional Radiology treatments stemmed from a collaborative approach involving a multidisciplinary tumor board composed of Radiologists, Interventional Radiologists, Gastroenterologists, Hepatologists, Hepato-Pancreato-Biliary (HPB) Surgeons, Oncologists, and Pathologists.

Treatment decisions for HCC considered not only the cancer stage but also the patient’s overall health, including frailty, other illnesses, tumor location, liver function, and potential treatment complications. Resource availability was also factored in to ensure a feasible treatment plan. This comprehensive approach, integrating these factors with the BCLC staging system and the concept of therapeutic hierarchy, allowed for personalized treatment strategies prioritizing therapies with the best chance of improving survival and minimizing side effects [29,30,31,32].

### 2.1. Procedures’ Protocol

Pre-intervention imaging and patient data are carefully evaluated by the Interventional Radiologist to rule out contraindications according to the Cardiovascular and Interventional Radiological Society of Europe (CIRSE) standard of practice documents [3,33].

**Figure 1:**
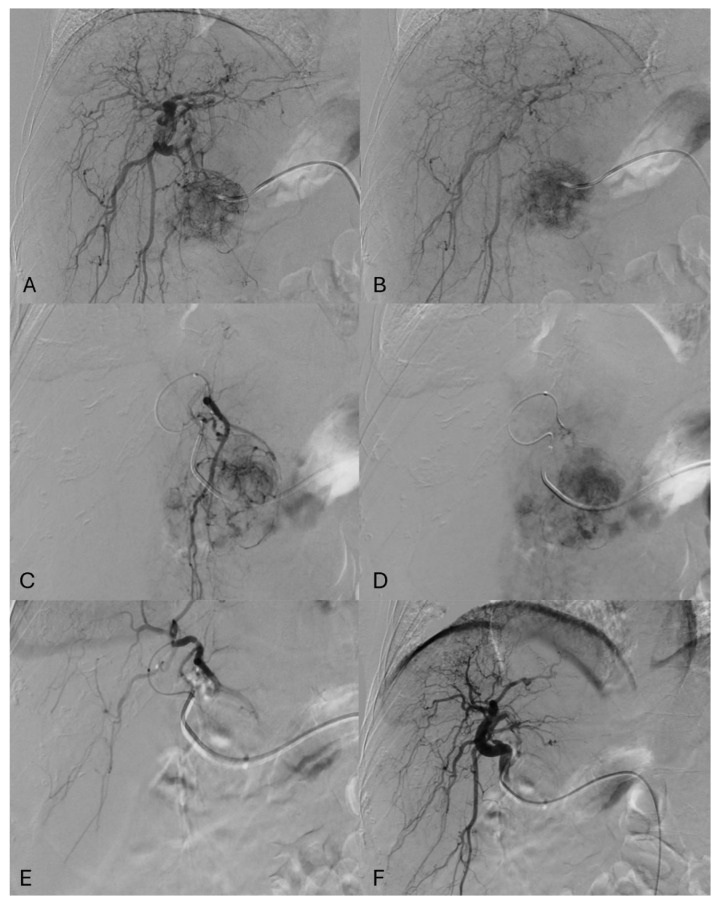
(**A**) Digital subtraction angiography (DSA), performed with Cobra C2 catheter tip (Imager II, Boston Scientific Corporation, Marlborough, MA, USA) positioned inside the proper hepatic artery with automatic injector (12 mL, 3 mL/s, 700psi, Iomeron 350, Bracco, IT), shows hepatic arterialization with anarchic hepatic nodule hypervascularization with characteristic “basket sign” pattern. (**B**) DSA at late arterial phase shows wash-in with retention of contrast media inside the hepatic nodule. (**C**,**D**) DSA shows superselective angiograms obtained using a microcatheter (Progreat 2.4, Terumo Inc., Tokyo, Japan). Here, each vessel supplying the HCC nodule is selectively catheterized, and microspheres (Embogold 100–300 micron, MERIT Medical System, South Jordan, UT, USA) are used to obstruct (embolize) these arteries. (**E**) shows a DSA from microcatheter demonstrating minimal to no blood flow in feeders vessels of HCC nodule. (**F**) the final DSA performed with the tip of the Cobra C2 catheter at the level of the proper hepatic artery confirms the procedure’s success by showing effective embolization as no contrast media reaches the anarchic vasculature of the HCC nodule. Following TAE or MWA, patients undergo meticulous monitoring to ensure successful outcomes. This typically involves a structured post-procedural care plan. Blood tests repeated 24 h and 72 h after the procedure check for abnormalities in blood cell counts, liver function, and kidney function—potential indicators of complications. Outpatient clinic visits are scheduled regularly, often starting one month after the procedure. At one month, blood tests and MRI are performed to evaluate the treated area and assess treatment tumor response.

In our Hospital, Interventional Radiologists perform TAE using calibrated microspheres without the use of chemotherapeutic agents [Figure 1] [34]. Cone-beam CT (CBCT) and vessel detection software are used on a standard base to pinpoint tumor feeder vessels and avoid, not target, embolization, by recognizing typical angiographic appearance. As no superiority in terms of survival benefit of other embolization techniques, such as DEB-TACE, C-TACE, and others, has been demonstrated, TAE is taken into account along with the operator’s preference and center experience [35,36,37].

The Interventional Radiology service uses MWA ablation as standard treatment in the HCC scenario (HS Hospital Service, Rome, Italy). In ablation cases, the choice of Microwave ablation technology is due to its ease of use, reproducibility, and larger volume of ablation, together with short procedural times that reduce the anesthesiologist’s support when requested [33,38].

According to our IR service treatments protocol, all patients undergoing HCC treatments perform pre-procedure lab tests with standard blood panel, liver function and clotting tests within the 5 days before the planned procedure. The patients planned for TAE or MWA are admitted to the hospital on the day of the procedure. Informed consent was obtained from all individual participants included in the study [39].

All subjects underwent MRI utilizing a 1.5-Tesla system, specifically the Signa HDxT (GE Healthcare, Milwaukee, WI, USA). The standardized institutional imaging protocol comprised 5 mm thickness dual-echo T1-weighted gradient-echo sequences, encompassing in-phase and out-of-phase images, navigator-triggered single-shot or multishot T2-weighted sequences, and diffusion-weighted (DW) images, with b values of 0, 50, 400, and 800 s/mm^2^, respectively. Dynamic imaging sequences were acquired with the administration of 10 mL of gadoxetic acid (Primovist, Bayer Schering Pharma, Berlin, Germany) at a rate of 1 mL/s, succeeded by a 20-mL 0.9% saline chaser at the same flow rate. The timing for the arterial phase was ascertained through either the test bolus or bolus-tracking technique. Subsequent imaging phases, namely portal venous phase, late portal venous phase, transitional phase (TP), and hepatobiliary phase (HBP), were acquired at approximately 60, 90, and 150 s, respectively, as well as 15–20 min post-contrast agent injection.

### 2.2. Evaluation and Inter-Reader Agreement Data Collection

In a blind assessment design, two board-certified abdominal radiologists with comparable levels of expertise (5 years of experience and 9 years of experience) independently evaluated the index lesions on post-LRT MRI. The readers were informed about the LRT methods and the time interval between LRT and the index MRI. They were granted access to pre-LRT imaging data, patient history, and relevant treatment reports, to facilitate a comprehensive assessment. The readers assessed each index lesion according to LR-TRA (LR-TR Viable, LR-TR Equivocal, or LR-TR Nonviable). The evaluation also considered the fact that ancillary features have been demonstrated which may improve tumor viability assessment [8,9,10].

The readers’ observations were meticulously documented on a standardized informatics datasheet in a structured format [40]. To further analyze the inter-reader variability and refine the assessment process, a revision of the initial evaluations and observations was undertaken. This revision facilitated the creation of subgroups based on two key criteria:

- Treatment modality: Patients were categorized according to the specific treatment they received, MWA and TAE.
- LI-RADS categorization concordance: Patients were further subdivided based on whether the two readers’ initial LR-TR gradings agreed (“matched”) or discordant (“mismatch”).

A blind adjudication process ensured a definitive categorization for cases with reader discrepancies. An independent, board-certified abdominal radiologist, unaware of the interpretations by the first two readers, reviewed the relevant data. This “reference standard” approach ensured a final, unbiased categorization for discrepant cases.

A comprehensive review and data collection of patient blood panels was conducted at baseline (T0), 3 days post-procedure (T1), and 30 days post-procedure (T2). For each patient, the Neutrophil-to-Lymphocyte Ratio (NLR) was calculated using a dedicated online tool (https://www.mdcalc.com) to ensure consistency. To minimize errors, a random sample of NLR calculations was rechecked for accuracy.

This approach provides valuable insights into potential associations between NLR and treatment type and inter-reader variability in LI-RADS assessment.

### 2.3. Statistical Analysis

Data were collected at baseline (T0), after 3 days of treatment (T1), and after 30 days of treatment (T2). Descriptive statistics included frequencies and proportions for categorical variables and mean ± standard. The agreement between readers in assigning the treatment response (LR-TRA) category by readers was evaluated by the Bangdiwala plot and Cohen’s Kappa (κ), interpreted according to the Landis and Koch scale. The *t*-test and the χ^2^ tests were used to assess differences between unpaired groups for continuous and categorical variables, respectively. Differences in the mean values of NLR by group over time were assessed by two-way mixed analysis of variance (Two-way ANOVA). All statistical tests were two-sided, with a significance level set at *p* < 0.05. Analyses were performed using the R software environment for statistical computing and graphics (version 4.1; http://www.r-project.org/).

## 3. Results

### 3.1. Study Population

A detailed review of interventional radiology procedural sheets identified 128 patients diagnosed with HCC who received either TAE or MWA within the defined study period.

**Figure 2:**
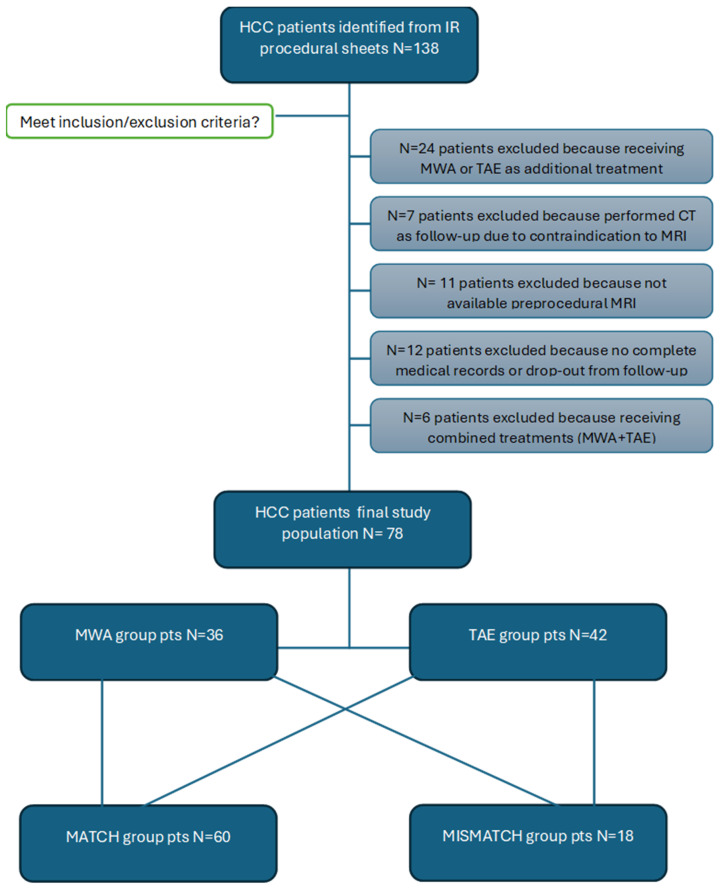
Flow chart diagram of study population and subgroups.

Following a rigorous application of pre-established inclusion and exclusion criteria, the final analysis included data from 78 patients. Figure 2 provides a flow diagram that visually depicts the process of patient selection and exclusion, clearly illustrating the number of patients identified, screened and ultimately included in the final analysis.

### 3.2. Baseline Data

A comprehensive overview of baseline patient characteristics is presented in Table 1. Patients were stratified by treatment modality: 36 received MWA and 42 underwent TAE. No statistically significant differences were found between the MWA and TAE groups regarding age, gender, Child-Pugh score, AFP levels, cirrhosis aetiology, or tumor size [Table 1].

### 3.3. LR-TRA Application

LR-TRA category assignment by the two independent observers reported:

LR-TR Non-Viable Category: A total of 32 lesions (41.2%) and 34 lesions (43.6%) were categorized as LR-TR non-viable by the first and second observers, respectively. Of these lesions, 18 (23.1%) and 22 (28.2%) for the first and second observer, respectively, underwent MWA, while the remaining lesions were treated with TAE (14/78; 17.9% and 12/78; 15.4%, respectively).

LR-TR Viable Category: A total of 41 lesions (52.6%) and 36 lesions (46.1%) were categorized as LR-TR viable by the first and second observers, respectively. MWA was performed on 15 lesions (19.2%) and 11 lesions (14.1%) based on the first and second observer classifications, respectively. The remaining lesions in this category (26/78; 33.3% and 25/78; 32.0%) were treated with TAE.

LR-TR Equivocal Category: A smaller proportion of lesions, 5 (6.4%) and 8 (10.2%) as categorized by the first and second observer, respectively, fell into the LR-TR equivocal category. MWA was used in 3 lesions (3.8% for each observer) based on these classifications, while the remaining lesions (2/78; 2.6% and 5/78; 6.4%) received TAE.

The external reviewer, possessing higher expertise in abdominal imaging, independently classified the observations as “non-viable” in 28 out of 78 cases (35.9%), “viable” in 45 out of 78 cases (57.7%), and “equivocal” in 5 out of 78 cases (6.4%). This independent evaluation achieved a high level of concordance (75.6%) with the consensus category assigned by the two primary readers. Interestingly, the agreement between the reviewers and the consensus reference was even greater within the treatment subgroups: 80.5% for the MWA scenario and 71.4% for the TAE scenario. These findings suggest a good level of overall agreement in LR-TRA category assignment.

### 3.4. Assessment of Inter-Reader Agreement

The level of concordance between the two independent radiologists in assigning LI-RADS treatment response (LR-TRA) categories to HCC lesions following interventional radiology treatments revealed agreement in 60 out of 78 cases (76.9%). This level of agreement was further evaluated statistically using Cohen’s Kappa (κ) coefficient, a robust measure for assessing inter-reader agreement in ordinal data.

**Figure 3:**
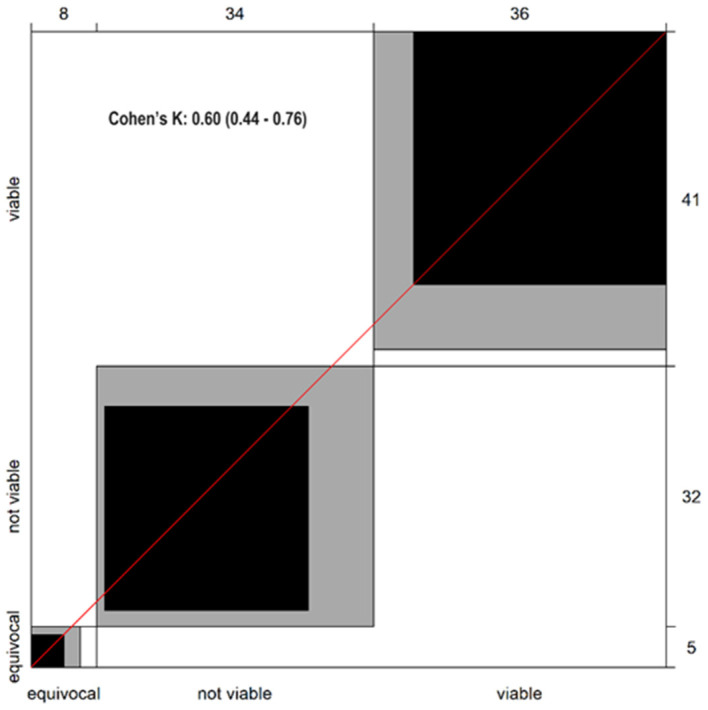
The general Inter-reader agreement graph plot for overall observations and Cohen’s K value. Bangdiwala plot visually represents agreement between two sets of categorical data using a square. The size of the black squares corresponds to the observed agreement for each category combination. The grey areas represent the maximum agreement possible given the marginal totals.

The calculated κ value of 0.60 indicates a “substantial” level of agreement according to the Landis and Koch interpretation scale. This finding suggests that the two readers demonstrated a high degree of consistency in classifying HCC lesions based on the LR-TRA criteria (Figure 3).

**Figure 4:**
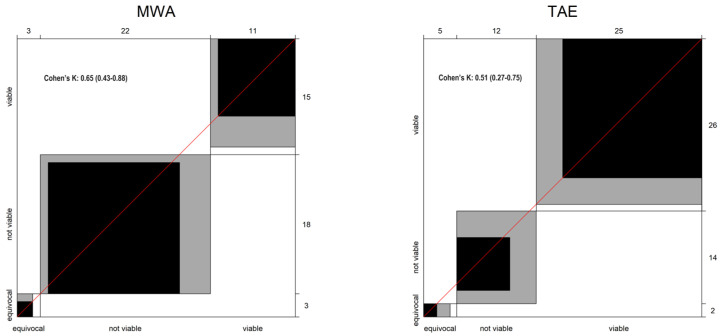
Inter-reader agreement graph plot and Cohen’s K value for MWA and TAE group patients’ observations.

For patients treated with MWA, the two readers demonstrated a high level of concordance in the LR-TRA application. Agreement was achieved in 29 out of 36 cases (80.5%). Statistical analysis using Cohen’s Kappa (κ) test yielded a value of 0.65, meaning “substantial” agreement according to the Landis and Koch scale (Figure 4). This finding suggests that the two readers consistently interpreted the LR-TRA criteria for MWA-treated lesions, potentially due to the well-defined treatment response patterns observed in this modality.

Conversely, the level of agreement for patients treated with TAE was slightly lower. The readers agreed on LR-TRA categorization in 31 out of 42 cases (73.8%). The κ coefficient for this subgroup was 0.51, indicating “moderate” agreement according to the Landis and Koch scale (Figure 4). This finding suggests that interpreting LR-TRA criteria for TAE-treated lesions might be more susceptible to reader variability. This may be attributable to the potentially heterogeneous response patterns observed following TAE compared to MWA.

The observed disparity in inter-reader agreement between MWA and TAE highlights the importance of considering treatment modality when interpreting LR-TRA classifications.

This study employed a Bangdiwala graph (Figure 3 and Figure 4) to visually represent the agreement between two independent radiologists in assigning LI-RADS treatment response (LR-TRA) categories to HCC lesions following ablative procedures. While the kappa coefficient is a widely used metric for inter-rater agreement, the Bangdiwala graph offers a complementary perspective by depicting the distribution of agreement across all possible category combinations.

### 3.5. NLR Evaluation

**Figure 5:**
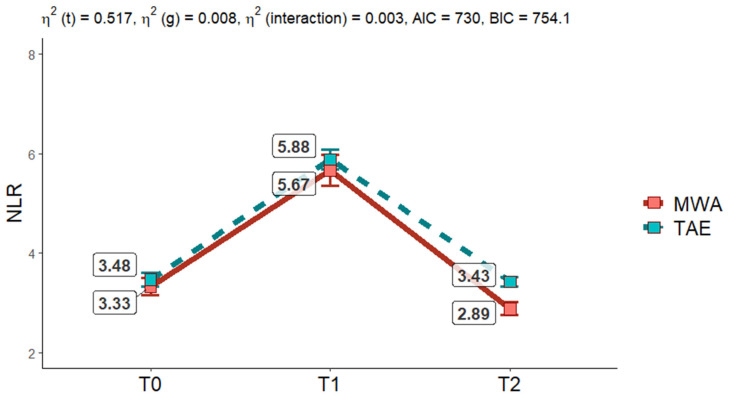
Two Way mixed ANOVA shows no statistically significative interaction between groups (*p* = 0.511); it demonstrates a statistically significant effect of time on NLR mean value changes in both groups (*p* ≤ 0.001) and differences in groups in NLR changes (*p* = 0.047).

Figure 5 illustrates the results of a Two-Way Mixed ANOVA assessing the dynamic changes in the NLR over time (T0 = baseline, T1 = 3 days after treatment, T2 = 30 days after treatment) in patients undergoing two locoregional treatments: microwave ablation (MWA) and transarterial embolization (TAE). This statistical model evaluates the main effects of time, group, and their interaction on NLR.

The analysis revealed a significant effect of time, indicating that NLR varied substantially across the three time points. At baseline (T0), NLR values were comparable between the two groups, reflecting a similar initial inflammatory state. Three days post-treatment (T1), there was a pronounced increase in NLR in both groups, with mean values peaking (MWA = 5.67; TAE = 5.88). This surge highlights a robust inflammatory response following both interventions. By one-month post-treatment (T2), NLR values decreased markedly, nearing baseline levels. However, a subtle difference emerged at this point, with the TAE group displaying slightly higher mean NLR values (TAE = 3.43; MWA = 2.89), suggesting a potentially slower resolution of inflammation compared to MWA.

The analysis also identified a significant effect of group, suggesting an overall difference in NLR between the two treatments, regardless of time. While this difference is statistically significant, the effect size is modest, indicating that the treatments may exert only slightly distinct inflammatory responses.

Importantly, the interaction between time and group was not statistically significant. This suggests that the pattern of NLR changes over time is consistent across both treatment groups, with no evidence of differential temporal dynamics between MWA and TAE.

**Figure 6:**
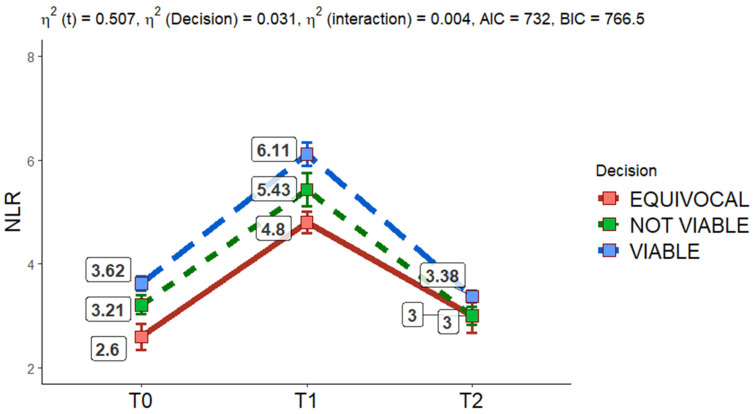
Two-way mixed ANOVA shows no statistically significant interaction between groups (*p* = 0.716); it demonstrates a statistically significant effect of time on NLR mean value changes in the different TRA categories (*p* ≤ 0.001) and differences in groups in NLR changes (*p* = 0.001).

The findings highlight the transient but vigorous nature of the inflammatory response induced by locoregional therapies, with a peak in NLR at T1 and a return to near-baseline levels by T2. Although both treatments follow similar temporal trends, the slightly higher NLR at T2 in the TAE group may point to subtle differences in the long-term resolution of inflammation. These results provide valuable insights into the inflammatory profiles associated with MWA and TAE, offering a deeper understanding of their post-treatment effects. Figure 6 illustrates the changes in NLR values across the different TRA categories (viable, not viable, equivocal), as determined by the reference standard third external observer category assignment.

The analysis reveals a statistically significant effect of time on NLR mean value changes (*p* < 0.001), indicating that the inflammatory response evolves markedly across the three-time points. Additionally, there is a statistically significant difference between the TRA categories (*p* = 0.001), suggesting that the magnitude of NLR changes varies across the groups. However, the interaction between time and group is not statistically significant (*p* = 0.716), indicating that the pattern of NLR changes over time remains consistent across the categories.

These findings highlight the transient but robust inflammatory response triggered by locoregional treatments, with a peak at T1 and resolution by T2. The subtle differences between categories underline the potential influence of treatment characteristics on the inflammatory trajectory.

### 3.6. TRA Discrepancy in Category Assignation and NLR

According to the result of the TRA application by the two independent readers, patients were divided into two subgroups. Group 1: patients with imaging evaluation existing in agreement between observers (MATCH group, 60 patients). Group 2: patients with imaging evaluation existing in disagreement between observers (MISMATCH group, 18 patients). The baseline data analysis of the MATCH group versus the MISMATCH group was conducted and demonstrated no statistically significant difference in tumor size, AFP level, Child-Pugh class, age or gender (Table 2).

**Figure 7:**
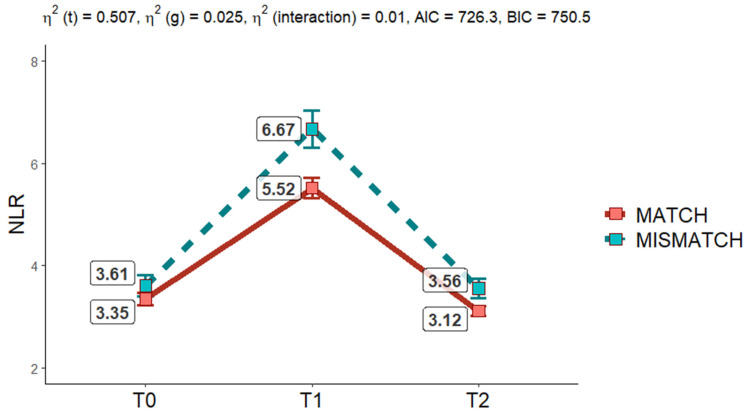
Interaction plot Two Way mixed ANOVA shows no statistically significative interaction between groups (*p* = 0.090); it demonstrates a statistically significant effect of time on NLR mean value changes in both groups (*p* < 0.001) and differences in groups in NLR changes (*p* < 0.001).

Figure 7 illustrates the dynamic changes in the neutrophil-to-lymphocyte ratio (NLR) over time (T0, T1, T2) across two groups: MATCH and MISMATCH, based on treatment alignment. The interaction plot, derived from a Two-Way Mixed ANOVA, reveals key findings regarding the temporal and group-level effects on NLR.

The analysis shows a statistically significant effect of time (*p* < 0.001, η^2^ (t) = 0.507), confirming that NLR values change markedly across the three time points. At baseline (T0), both groups exhibit comparable NLR levels (MATCH = 3.61, MISMATCH = 3.35). A significant increase is observed at T1, with the MISMATCH group reaching a higher peak (6.67) compared to the MATCH group (5.52). By T2, NLR levels decrease in both groups, approaching baseline values, though the MISMATCH group retains slightly higher levels (3.56 vs. 3.12).

There is also a significant effect of group (*p* < 0.00, η^2^ (g) = 0.025), indicating overall differences in NLR changes between MATCH and MISMATCH groups, with the latter consistently showing higher values. However, the interaction between time and group is not statistically significant (*p* = 0.090, η^2^ (interaction) = 0.01), suggesting that the temporal trend of NLR changes is consistent across both groups.

These results highlight a robust, transient inflammatory response, peaking at T1 and resolving by T2. The consistently higher NLR values in the MISMATCH group suggest a potentially more pronounced or prolonged inflammatory reaction, which may reflect differences in the underlying biological or treatment-related factors between the two groups.

## 4. Discussion

Our investigation aimed to delve into the inter-reader agreement in the application of the Tumour Response Algorithm (LR-TRA) and its association with the Neutrophil-to-Lymphocyte Ratio (NLR) in patients treated with TAE versus MWA for hepatocellular carcinoma (HCC). Our findings shed light on the reliability of LR-TRA in evaluating tumor response and its potential interplay with NLR dynamics in HCC patients undergoing distinct treatment modalities.

In our research, the inter-reader agreement in LR-TRA assessment was discovered to be “substantial” in MWA patients and “moderate” in TAE patients. This implies that LR-TRA is a more dependable tool for evaluating tumor response in MWA patients compared to TAE patients. It suggests that the type of interventional radiology procedure employed for HCC treatment significantly influences the reliability and interpretation of LR-TRA assessments. This could have significant implications for patient care, as it could mean that TRA values obtained from patients undergoing different treatment modalities may have varying accuracy. Technique-related factors may be one of the causes of the discrepancy in LR-TR application and performance. MWA is a more operator-independent technique compared to TAE, which could lead to more consistent and reproducible tumor ablation patterns. This consistency could facilitate a more straightforward assessment of tumor response using LR-TRA as demonstrated by our results.

Although the LR-TR was created to evaluate treated HCC regardless of the locoregional treatment used, the post-treatment imaging features are treatment-specific, which could lead to variations in reader agreement.

A confluence of factors may contribute to the heterogeneity of data available in the literature about the inter-reader agreement in TRA and the performance of LR-TR. For instance, the imaging modality of TR evaluation also constitutes a key element of heterogeneity. In the interpretation of CT after Transarterial Chemoembolization (TACE), highly dense iodized oil deposition can hinder accurate assessment by directly obscuring enhancement in the viable tumor portion or indirectly obscuring it through beam-hardening artefacts [41,42], potentially diminishing the reliability of the imaging interpretation. In contrast, iodized oil barely obscures viable HCC on MRI [43,44]. However, particularly with the use of gadoxetic acid as a contrast agent, the benefits of MRI may be counteracted by the weak arterial-phase hyperenhancement, due to the relatively small contrast dose and strict washout criteria that are confined to the portal venous phase according to the current LI-RADS [45]. In our study, we used MRI-only imaging to obtain the best support from imaging analysis and avoid other factors that may influence LR-TR inter-reader agreement.

Studies used various imaging modalities like CT or MRI to assess treatment response, potentially contributing to variability in inter-reader agreement. The specific treatment modality, such as TAE, RFA, MWA, C-TACE, or DEB-TACE, might also influence agreement, as some treatments may produce more easily recognizable imaging changes. Additionally, some studies lacked comparative data on inter-reader agreement between different local-regional therapies, making it difficult to draw definitive conclusions about which therapies have the most consistent interpretation of treatment response [6,7,8,9,10,11,12,46,47,48].

Our study goes beyond these previous investigations by employing a more rigorous methodological approach. First, as already pointed out, we exclusively utilized MRI for response evaluation, eliminating the variability inherent in using different imaging techniques. This focus on a single, high-quality imaging modality enhances the accuracy of our inter-reader agreement assessment. Second, we directly compared two standardized treatment modalities, MWA and TAE, with specific institutional protocols, minimizing potential confounding factors and allowing for a more precise analysis. Finally, and most notably, our study is the first to investigate the influence of inflammation, as reflected by the dynamic changes of NLR values, on inter-reader agreement. This novel finding introduces a new dimension to our understanding of the factors that contribute to variability in treatment response assessment and has not been previously explored in this context.

The NLR represents a marker of systemic inflammatory response and has emerged as an independent prognostic biomarker for patients with cancer [28]. The host’s inflammatory response to cancer and/or the systemic effects mediated by cancer cells can induce the upregulation of the inflammatory response [49]. Elevated systemic inflammation, as reflected by the NLR, has been associated with cancer progression, metastasis, and poor clinical outcome in various tumors. Its use as a prognostic biomarker of OS in patients with HCC has been largely demonstrated in the literature but its dynamic changes after locoregional therapies remain underexplored. This knowledge gap stems from the complexity of immune responses to these treatments, the emerging interest in autoimmunity response to tumor, and the heterogeneity of existing research. In this context, our study investigates NLR dynamics after MWA and TAE for HCC, aiming to shed light on this understudied area and its implications for treatment response and patient outcomes.

The dynamic change of NLR proved to be specular in the MWA group and TAE group, demonstrating that LRTs trigger an important immune response. In our series, NLR surge at T1 (3 days) was demonstrated to be more consistent in patients treated with TAE, with a higher mean NLR value persisting at delayed evaluation. These data, matched with the resulting difference in inter-reader agreement, may be interpreted as a higher value of NLR may be associated with a difficulty in imaging interpretation and a higher level of disagreement between observers. One of the discriminants may be found in analyzing the different immunologic responses in MWA and TAE treatments.

In the case of MWA ablation, in the necrotic microenvironment, an increase in T cells and IL-12 is demonstrated to be triggered by immunogenic cell death. The high temperature generated by the MWA probe results in an inflammatory response with low IL-6 and neutrophil immunomodulation [50]. The MWA directly destroys tumor cells and the coagulation necrosis zone around the ablation site can act as a physical barrier to angiogenesis. The hypervascularity after treatments induced by VEGF seems to be transient.

Conversely, in TAE treatment, even if treatment is technically successful, the blood supply to the tumor is only virtually blocked. This may result in tumor cells continuing to release angiogenic factors that promote the formation of new blood vessels. Moreover, inflammatory change due to blood restriction and necrosis may favor the neutrophilia that may act to suppress the cytotoxic activity of immune cells, such as activated T cells and natural killer cells, as documented in numerous studies [50]. Additionally, clinical investigations of HCC patients revealed that peritumoral and intratumoral neutrophils were positively correlated with angiogenesis progression and VEGF expression [51,52].

The complex inflammatory microenvironment fostered by vasogenic factors and cytokines creates conditions that can induce errors in observer tumor response interpretation as blood flow and inflammatory changes at the treatment site may mimic the appearance of viable tumors on imaging studies.

The NLR mean value was demonstrated to be higher in patients with HCC lesions interpreted at imaging as “viable” according to LR-TRA as demonstrated in Figure 6 This data is in line with the literature that considers NLR as a prognostic marker and its high value as a predictor of recurrence [53].

Future research could focus on larger and multicenter studies needed to corroborate the observed association between inter-reader agreement, NLR levels, and treatment modality in a more generalizable setting. This is particularly important as the current study does not establish causality due to its inherent limitations. Longitudinal studies could track NLR and LR-TRA changes over time to assess their dynamic relationship and identify potential prognostic markers. Examining the correlation between NLR levels, inter-reader agreement discrepancies, and clinical outcomes such as tumor progression, survival and adverse events could further elucidate the clinical significance of this biomarker.

Our study has some limitations; the retrospective nature of the study, coupled with the relatively modest sample size, necessitates circumspection in extrapolating the findings to a broader patient population. The small sample size still feels the effect of the COVID-19 scenario as part of the study considering a time frame that was largely influenced by difficulties in the offer of proper treatments to oncologic patients during the pandemic. Moreover, the focus on just two treatment modalities could have inadvertently omitted alternative treatment association options, potentially limiting the comprehensiveness of the evaluation. Additionally, the brevity of the follow-up period might have precluded a thorough assessment of long-term tumor response and its implications for treatment efficacy. In addition, the reliance on two readers for inter-reader agreement evaluation might have introduced a degree of variability in image interpretation, highlighting the need for further validation studies with a larger cadre of readers. We acknowledge the potential for confirmation bias. To mitigate this, readings were performed independently by two radiologists with comparable expertise from different training institutions, and kappa statistics were used. Future studies could include a more diverse reader pool to further minimize bias. Finally, we recognized the potential for bias introduced by relying on a single adjudicator for resolving inter-reader discrepancies, and we took several measures to mitigate this risk. Firstly, the external reviewer, with over 15 years of experience in abdominal imaging, was blinded to the initial readings and clinical data, minimizing potential influence from prior assessments. Secondly, adherence to the standardized LR-TRA criteria ensured a consistent and objective approach to image interpretation. However, recognizing that a single adjudicator, even with extensive expertise and a blinded process, may still introduce some degree of subjectivity, future studies with larger sample sizes could incorporate multiple adjudicators to further enhance the objectivity of the discrepancy resolution process.

The findings of this study have significant implications for both patient care and research in the field of HCC treatment. The identification of a lower inter-reader agreement for LR-TR scores in TAE patients compared to MWA highlights the need for more meticulous interpretation and monitoring of LR-TR in TAE patients. While this study utilized a comprehensive MRI protocol, further refinements could enhance the assessment of TAE-treated HCC; incorporating high-resolution 3D T1-weighted sequences and optimizing the timing of hepatobiliary phase acquisition could improve lesion detection and characterization. Additionally, advanced techniques like perfusion imaging and the development of standardized protocols, such as those promoted by QIBA, hold promise for reducing inter-observer variability. The integration of AI algorithms could further enhance image analysis and interpretation [54].

Furthermore, the potential of NLR to serve as a predictive biomarker for inter-reader discrepancies in LR-TR assessment suggests a novel approach to identifying patients at risk of assessment difficulties and potentially requiring closer monitoring.

Combining the assessment of LR-TRA and NLR levels could provide a more comprehensive understanding of tumor response and inflammatory dynamics in HCC patients undergoing TAE or MWA. This could effect more informed treatment decisions and optimize patient outcomes.

The study findings also contribute to a growing body of evidence supporting the role of NLR as a biomarker for HCC treatment response and inflammation.

The development of reliable and clinically actionable biomarkers for HCC treatment response and inflammation holds immense promise for enhancing patient care and improving patient outcomes leading to personalized treatment strategies tailored to individual patient characteristics and inflammatory profiles.

## 5. Conclusions

In conclusion, this study has provided valuable insights into the reliability of LR-TRA for assessing tumor response in HCC patients undergoing TAE or MWA. The potential of NLR as a predictive biomarker for inter-reader discrepancies and the combined assessment of LR-TRA and NLR for a more comprehensive assessment of tumor response and inflammatory dynamics offer promising avenues for future research and clinical practice.

## Figures and Tables

**Table 1:** Clinical and Demographic baseline patient characteristics of the two groups (MWA and TAE) are expressed as mean ± standard deviation for continuous variables and absolute frequency (column percentage) for categorical. MWA = microwave percutaneous liver ablation, TAE = transarterial embolization.

Variables	Overall (N = 78)	MWA (N = 36)	TAE (N = 42)	*p*-Value
**Gender, n (%)**				0.411
Male	45 (57.7)	19 (52.7)	26 (61.9)	
Female	33 (42.3)	17 (47.3)	16 (38.1)	
**Age, years**				0.412
Mean	74.1 ± 10.1	73.2 ± 10.3 years	75.1 ± 9.7 years	
Min	49	49	53	
Max	93	93	89	
**Cirrhosis aetiology, n (%)**				0.960
Viral	41 (52.6)	19 (52.7)	22 (52.4)	
Alcohol	23 (29.5)	11 (30.6)	12 (28.6)	
NASH/other	14 (17.9)	6 (16.7)	8 (19.0)	
**Child-Pugh, n (%)**				0.961
A	64 (82.0)	31 (86.1)	33 (78.6)	
B	14 (18.0)	5 (13.9)	9 (21.4)	
**Alpha-fetoprotein, n (%)**				0.200
<400 ng/mL	32 (41.0)	14 (38.9)	18 (42.8)	
>400 ng/mL	46 (59.0)	22 (61.1)	24 (57.2)	
**Tumour size, cm**				0.080
Mean	3.14 ± 0.53	3.03 ± 0.46	3.23 ± 0.56	
Max	4.5	4.2	4.5	
Min	2.0	2.2	2.0	

**Table 2:** Baseline characteristics of patients in the MATCH (n = 60) and MISMATCH (n = 18) groups. Statistical comparisons were performed using the chi-square test for categorical variables and the independent samples *t*-test for continuous variables. Data are presented as mean ± standard deviation, minimum, and maximum values or as counts and percentages.

Variables	MATCH (N = 60)	MISMATCH (N = 18)	*p*-Value
Gender, n (%)			0.061
Male	38 (63.3%)	7 (38.9%)	
Female	22 (36.7%)	11 (61.1%)	
Age, years			0.523
Mean	73.7 ± 9.1 years	73.8 ± 13.1 years	
Min	49 y.o.	53 y.o.	
Max	93 y.o.	89 y.o.	
Cirrhosis aetiology			0.623
Viral	30 (50%)	11 (61.1%)	
Alcohol	18 (30%)	5 (27.8%)	
NASH/other	12 (20%)	2 (11.1%)	
CHILD-PUGH			0.962
A	49 (81.7%)	15 (83.3%)	
B	11 (18.3%)	3 (16.7%)	
Alpha-fetoprotein			0.151
<400 ng/mL	22 (36.7%)	10 (55.6%)	
>400 ng/mL	38 (63.3%)	8 (44.4%)	
Tumour size			0.161
Mean	3.18 ± 0.56 cm	2.98 ± 0.35 cm	
Max	4.5 cm	4 cm	
Min	2 cm	2.5 cm	

## Data Availability

The raw data supporting the conclusions of this article will be made available by the authors on request.
